# Genome-wide CRISPR interference screen identifies *Clip2* as a novel regulator of osteocyte maturation and morphology

**DOI:** 10.1016/j.jbc.2026.113075

**Published:** 2026-04-27

**Authors:** Courtney M. Mazur, Parthena E. Kotsalidis, Majd George, Tom Whalley, Tadatoshi Sato, John G. Doench, Lauren E. Surface, Marc N. Wein

**Affiliations:** 1 Endocrine Unit, Massachusetts General Hospital, Harvard Medical School, Boston, Massachusetts, USA; 2 Wellcome Centre for Cell-Matrix Research, Faculty of Biology, Medicine and Health, University of Manchester, Manchester, UK; 3 Division of Rheumatology, Department of Medicine, University of Massachusetts Chan Medical School, Worcester, Massachusetts, USA; 4 Broad Institute of MIT and Harvard, Cambridge, Massachusetts, USA; 5 Department of Biologic and Materials Sciences & Prosthodontics, University of Michigan School of Dentistry, Ann Arbor, Michigan, USA

**Keywords:** genome-wide CRISPR screen, osteocyte, cytoskeleton

## Abstract

Osteocytes play critical roles in bone, making them attractive targets for therapeutics aimed at improving bone mass and strength. The genes driving osteocyte maturation and function are not fully understood. Here, we aimed to identify novel genes responsible for osteocyte differentiation and dendrite development by performing a genome-wide CRISPR-interference (CRISPRi) screen in the Ocy454 osteocyte-like cell line. We identify CD61 (integrin β3) as a marker of osteocyte maturation: surface CD61 expression increases during osteocyte maturation, and CD61^high^ cells express higher levels of osteocyte marker genes. We then developed a flow cytometry-based assay to quantify surface CD61 protein levels as a phenotypic endpoint for functional genomic screening. In a genome-wide screen, we identified *Clip2*, which encodes a microtubule-binding protein, as one of dozens of genes necessary for CD61 expression. *Clip2* inhibition decreased surface CD61 expression, reduced expression of osteocyte-specific genes *Dmp1* and *Sost*, and impaired dendrite morphology *in vitro*. Together, these results highlight the utility of surface CD61 as a marker of osteocyte maturity and identify the role of the microtubule cytoskeleton for osteocyte differentiation, form, and function.

Osteocytes play critical roles in local mineral remodeling, mechanosensation, bone cell coordination, and endocrine signaling (1, 2). Ablation of osteocytes leads to porous, fragile bone (3). As osteocytes are post-mitotic, long-lived cells, proper differentiation and morphogenesis have long-term effects on osteocyte function and bone strength. Osteocyte differentiation involves transcriptional and morphologic changes as osteoblasts embed into bone. Although marker genes expressed at different stages of osteoblast-to-osteocyte differentiation have been described (4, 5, 6), the genes that drive osteocyte differentiation and mature morphology remain largely unknown.

Key among the defining characteristics of osteocytes are the numerous dendritic projections extending from each cell body. Dendrites extend through a network of canals in bone, allowing connection to nearby cells *via* gap junctions (7, 8). They also massively increase the surface area of osteocytes and house mechanosensitive structures that respond to bone loading (9, 10). Previously, osteocyte dendrites have been described primarily as actin-rich and actin-dependent structures, with short-term depolymerization of microtubules having little effect on dendrite morphology (11, 12). However, microtubules contribute to cytoskeletal tension and mechanosensitivity in osteocytes (13, 14, 15), and the role of tubulins and intermediate filament proteins in the extension and long-term maintenance of osteocyte dendrites has not been explored. This leaves open questions regarding the roles of cytoskeletal proteins and their binding partners in establishing osteocyte dendrites and maintaining their functional phenotypes.

Recent work has aimed to define an osteocyte-specific transcriptome that highlights the differences between osteocytes and other cells in the osteoblast lineage (5, 6, 16). However, the transcriptional changes that drive osteocyte differentiation and acquisition of their dendritic morphology have not been studied at the genome-wide level. Genome-wide clustered regularly interspaced short palindromic repeats (CRISPR)-mediated screens are a high-throughput strategy to measure the effects of individual genetic perturbations on cell viability or phenotype (17). Optimized and modified Cas9 enzymes now allow for gene ablation through double-strand breaks (18), transcriptional inhibition and activation (19), and base editing (20). Pooled CRISPR-based screens have been rapidly adopted to uncover genes controlling drug resistance (17), cell proliferation & maturation (21), protein trafficking (22, 23), and cell shape (24), but genome-wide screens investigating the osteogenic lineage (21, 25, 26, 27) have not studied differentiation beyond osteoblasts.

Here, we sought to identify novel genes responsible for osteocyte differentiation and dendrite development by performing a genome-wide CRISPR-interference (CRISPRi) screen in the Ocy454 osteocyte-like cell line. We developed a flow cytometry-based assay to quantify surface CD61 as a marker of osteocyte maturation and validated our screening results through quantification of osteocyte-specific gene expression and dendrite morphology *in vitro.* Together, these results highlight the utility of surface CD61 as a marker of osteocyte maturity and identify a role of the microtubule cytoskeleton in osteocyte differentiation, form, and function.

## Results

### CD61 is a marker of osteocyte maturity

**Figure 1:**
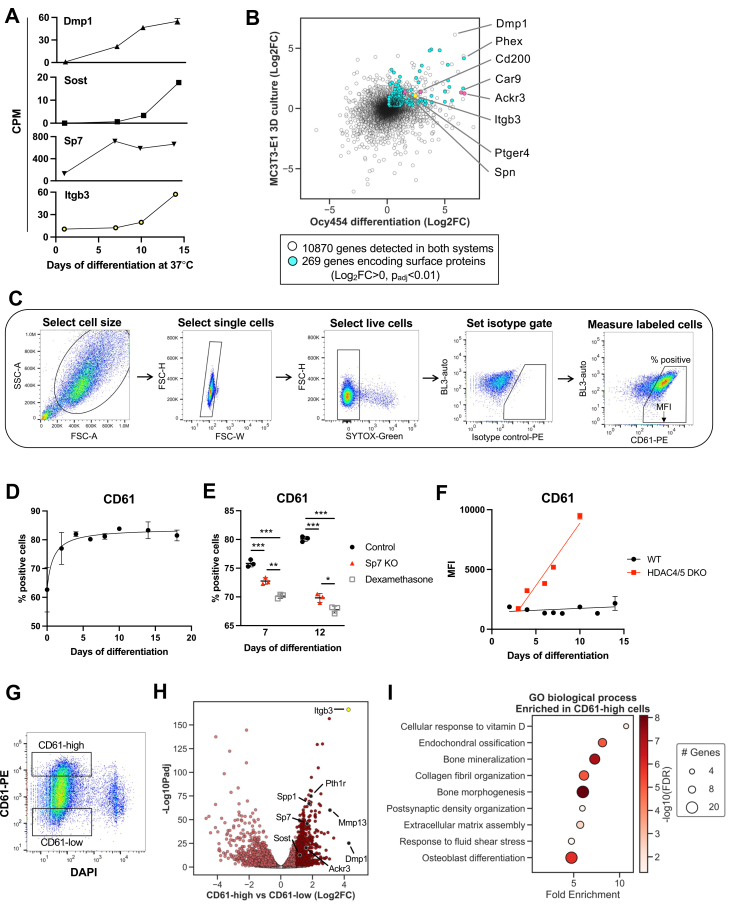
**Identification of CD61 as a marker of osteocyte differentiation.***A*, counts per million (CPM) for select osteoblast-osteocyte lineage marker genes from day 1 to day 14 of Ocy454 differentiation in 2D culture by RNA-sequencing. Error bars show standard deviation for two biologic replicates at each time point. *B*, scatter plot of genes expressed in Ocy454 cell differentiation (x-axis: Day 14 *versus* Day 1) and MC3T3-E1 cell 3D *versus* 2D culture (y-axis). Genes upregulated in both datasets that encode surface proteins are plotted in *cyan* in the *upper right* quadrant. Genes encoding candidate flow cytometry markers are plotted in *magenta*. *Itgb3* (plotted in gold, encodes CD61) was ultimately selected. *C*, flow cytometry gating strategy for CD61 detection in live Ocy454 cells. Percent CD61-positive cells and CD61-PE median fluorescence intensity (MFI) are calculated from single, live (SYTOX-green-negative) cells. *D*, Flow cytometry results show percent of single, live cells labeled with CD61-PE antibody compared to isotype control at a range of differentiation timepoints for wild-type Ocy454 cells. Error bars show standard deviation for all replicates at each time point. *E*, flow cytometry results show percent of single, live cells labeled with CD61-PE antibody compared to isotype control for wild-type Ocy454 cells, *Sp7* knockdown Ocy454 cells, and wild-type Ocy454 cells treated with 1 μM dexamethasone for 24 h. Error bars show standard deviation for three biologic replicates at each time point. ∗*p* < 0.01, ∗∗*p* < 0.001, and ∗∗∗*p* < 0.0001 by two-way ANOVA followed by Sidak’s multiple comparisons tests between cell types at each timepoint. *F*, flow cytometry results show median fluorescence intensity (MFI) of CD61-PE in populations of single, live HDAC4/HDAC5 double knockout Ocy454 cells compared to wild-type Ocy454 cells at a range of differentiation timepoints. Error bars show standard deviation for all replicates of each cell type at each time point. Lines indicate simple linear regression for each cell type, *p* < 0.0001 between genotypes. *G*, flow cytometry gates show the single, live (DAPI-negative) cells with the 10% highest expression of CD61 (CD61^high^) and 10% lowest expression of CD61 (CD61^low^), collected by FACS for RNA-sequencing after 17 days of differentiation at 37 °C. *H*, Volcano plot of 12,088 genes detected in all FACS RNA-seq samples. 564 genes are significantly enriched in CD61^high^ cells with Log2FC > 1 and *p*_adj_ < 0.01, n = 3 per group. *I*, Top gene ontology terms enriched in 564 genes upregulated in CD61^high^ cells.

First, to perform a flow cytometry-based phenotypic CRISPR screen, we aimed to identify a surface marker to distinguish the differentiation state of osteocytes. We interrogated gene expression data from two different *in vitro* differentiation assays which capture, to some degree, the osteoblast-to-osteocyte transition: Ocy454 cells cultured at 37 °C for 14 days *versus* 1 day and MC3T3-E1 cells cultured in 3D collagen gels compared to growth in 2D on standard polystyrene tissue culture dishes. Ocy454 osteocyte-like cells are conditionally immortalized due to constitutive expression of the thermosensitive large T antigen. Thus, these cells divide at 33 °C and stop dividing and upregulate osteocyte-like gene expression patterns over time at 37 °C (Figs. 1*A*, S1, *A*–*D*, Data S1) (28). 3D culture of MC3T3-E1 osteoblastic cells induces growth of long dendrite-like cytoskeletal processes and an osteocyte-like transcriptome (5). Significant upregulation of osteocyte differentiation markers *Sp7*, *Dmp1*, and *Phex* was noted in both systems, and *Sost* was significantly upregulated during Ocy454 differentiation (Fig. 1, *A* and *B*).

From these RNA-sequencing datasets, we identified candidate cell surface proteins encoded by genes that were upregulated in both differentiation systems (*Spn*: CD43, *Ackr3*: CXCR7, *Car9*: CA-IX, *Cd200*: CD200, *Ptger4*: PTGER4, *Itgb3*: CD61) and measured their expression on Ocy454 cells by flow cytometry (Figs. 1*C*, S2, *A*–*D*). An ideal marker would be strongly upregulated over time at 37 °C in Ocy454 cells, detected at high levels on a large percentage of differentiated cells, convenient to use (detected with a commercially available fluorescently conjugated antibody), and physiologically relevant.

Of the candidates tested, integrin beta-3 subunit (*Itgb3,* CD61) best met these criteria (Figs. 1, *D*–*F*, S2*A*). The choice of CD61 as a differentiation marker is bolstered by its physiological relevance in osteocytes. Integrin beta-3 protein is found along osteocyte dendrites *in vivo* (29, 30), is required for a full mechanosensitive response (31, 32), and its expression can be modulated by bone-relevant stimuli. Dexamethasone treatment, a clinically relevant pharmacologic insult that reduces osteocyte viability (15, 33), reduces CD61 expression on Ocy454 cells (34) (Fig. 1*E*). Furthermore, Ocy454 cells lacking *Sp7*, a transcription factor needed for dendrite development (5), express less CD61 than WT cells (Fig. 1*E*). In contrast, Ocy454 cells lacking HDAC4 and HDAC5, which show accelerated differentiation with high expression of *Sost* (35), have increased CD61 expression (Fig. 1*F*).

Next, the physiologic significance of surface CD61 expression was explored by performing RNA-sequencing of CD61^high^ *versus* CD61^low^ Ocy454 cells after 16 days of differentiation at 37 °C (Fig. 1*G*). As expected, CD61^high^ cells show increased *Itgb3* expression *versus* CD61^low^ cells (Fig. 1*H*). Gene ontology analysis reveals that CD61^high^ cells are enriched for genes related to ossification, ECM organization, and cell adhesion, such as *Dmp1, Sost, Spp1, Mmp13, Sp7, Pth1r,* and *Ackr3* (Figs. 1, *H* and *I*, S3, Data S2). Together, these results support the choice of surface CD61 as a marker of osteocyte maturity for subsequent genome-wide screening.

### Design of a genome-wide CRISPRi screen in Ocy454 cells

**Figure 2:**
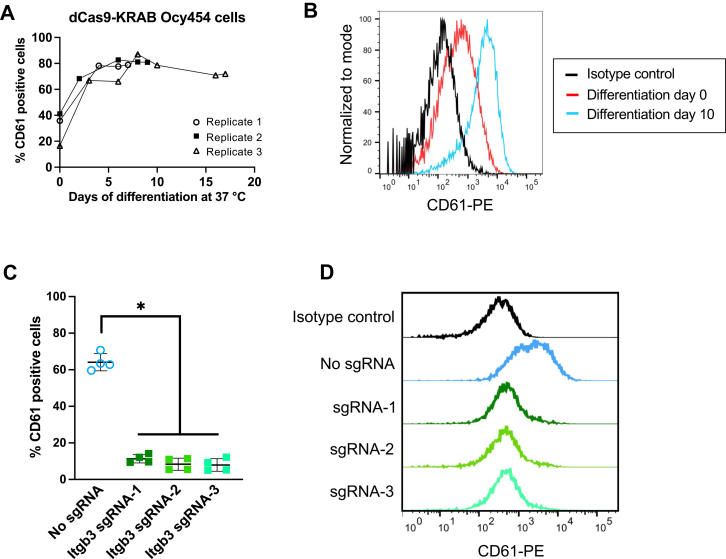
**CRISPRi-mediated CD61 silencing in Ocy454 cells.***A*, flow cytometry results show percentage of single, live dCas9-KRAB-expressing Ocy454 cells labeled with CD61-PE antibody compared to isotype control at a range of differentiation timepoints. Lines connect matched subcultures of cells followed over time. Error bars show standard deviation for two technical replicates in each test. *B*, histograms show intensity of CD61-PE fluorescence compared to isotype control for populations of single, live Ocy454 cells differentiated for the indicated times. *C*, flow cytometry results show percent of single, live dCas9-KRAB-expressing Ocy454 cells labeled with CD61-PE antibody compared to isotype control in cells with no sgRNA and cells expressing sgRNAs targeting the *Itgb3* promoter at differentiation day 11. Each point represents one biological replicate. Error bars show standard deviation. ∗*p* < 0.0001 by one-way ANOVA followed by Dunnett’s multiple comparisons tests *versus* no sgRNA control. *D*, histograms show intensity of CD61-PE fluorescence compared to isotype control for populations of single, live Ocy454 cells expressing sgRNAs targeting the *Itgb3* promoter.

We aimed to identify genes that, when inhibited, interfere with Ocy454 cell maturation, detected as reduced CD61 surface expression. Due to concerns that double-stranded DNA breaks might affect cell proliferation and differentiation in this long-term assay (36), we chose to perform a CRISPR-interference (CRISPRi) screen. We transduced Ocy454 cells with a BFP-expressing lentivirus expressing nuclease-deficient Cas9 (dCas9) fused with transcriptional repressor KRAB (19, 37), and sorted twice for BFP^high^ cells. Similar to parental cells, the resulting “dCas9-Ocy454 cells” upregulated CD61 between 0 and 10 days of differentiation (Fig. 2, *A* and *B*). To demonstrate the functionality of the dCas9 system, we introduced three unique sgRNAs targeting the *Itgb3* promoter. After 11 days of differentiation, all three sgRNAs reduced the percentage of CD61-positive cells from approximately 70% to 10% (Fig. 2, *C* and *D*).

The percentage of cells labeled and labeling intensity of CD61 was insensitive to the volume of antibody used between 0.25 and 2 μl per 1,000,000 cells (Fig. S4*A*). As Ocy454 cells differentiate in 2D culture, they produce an extracellular matrix that must be digested to dissociate cells into a single-cell suspension for flow cytometry. We compared multiple cell dissociation enzymes and found that trypsin resulted in lower median fluorescence intensity of CD61-PE than the other enzymes tested (Fig. S4*B*). However, while all enzymes tested were sufficient to liberate undifferentiated osteocytes, highly concentrated Trypsin-EDTA yielded the most live differentiated cells for analysis (Fig. S4*C*). This made carefully timed application of Trypsin-EDTA the preferred method for liberation of matrix-embedded Ocy454 cells for flow cytometry analysis.

### CRISPRi screen

**Figure 3:**
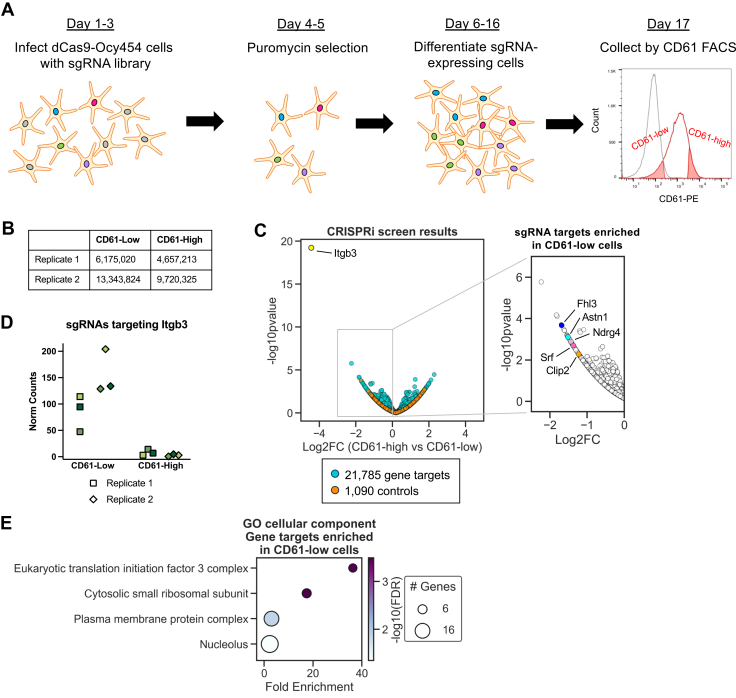
**Overview of CRISPRi screening.***A*, schematic of CRISPRi screen timeline. Histogram represents one replicate of single, live dCas9-Ocy454 cells labeled with CD61-PE antibody (*red line*) or isotype control (*grey line*). *Red shaded* areas indicate the lowest and highest 10% of cells based on CD61-PE intensity. *B*, number of cells collected in CD61^high^ and CD61^low^ groups in each replicate of the CRISPRi screen. *C*, Volcano plot shows CRISPRi screen results for CD61^high^*versus* CD61^low^ groups for both replicates. Each point represents one gene targeted by at least 3 sgRNAs in the pooled screening library. Control points represent groups of three non-targeting or intergenic site-targeting sgRNAs. Inset shows all gene targets enriched in CD61^low^ cells (Log2FC < 0) with top candidates for follow-up labeled. *D*, normalized counts of all sgRNAs targeting the *Itgb3* promoter detected in each replicate of the CRISPRi screen. Colors denote each of three sgRNAs. *E*, Top gene ontology terms from 202 genes targeted by sgRNAs enriched in CD61^low^ cells.

For screening, we initially aimed to express each of 66,000 sgRNAs in the pooled library in 200 cells (200x representation). To avoid cells simultaneously infected with multiple sgRNA-expressing lentiviruses, we performed titer calculations to achieve lentiviral infection efficiency between 30 and 50% (Fig. S5, *A*–*C*). After pooled library infection and puromycin selection, the heterogeneous population of sgRNA-expressing dCas9-Ocy454 cells proliferated at 33 °C until confluent, then differentiated for 10 days at 37 °C. We labeled cells with CD61-PE antibody and collected live cells with the 10% highest and 10% lowest expression of CD61 (Fig. 3*A*). sgRNAs in the genomic DNA of each population were sequenced to identify putative genes that affected CD61 expression.

In each of the two independent replicates, 4.6 to 13.3 million cells per group were collected for gDNA isolation and sequencing (Fig. 3*B*). We compared CD61^high^ to CD61^low^ cells and identified 202 gene targets significantly depleted from CD61^high^ cells (enriched in the CD61^low^ group: Log_2_FC < −1, *p* < 0.05), representing genes that are necessary for high CD61 expression and osteocyte differentiation (Fig. 3*C*, Data S3). *Itgb3* was the most significantly enriched gene target in the CD61^low^ group in both replicates, which served as a robust positive control (Figs. 3, *C* and *D*, S5*D*). Genes encoding components of the translation initiation complex (*e.g. Eif3b*) and ribosomal subunit proteins (*e.g. Rpl18*) were also enriched in the CD61^low^ group (Figs. 3*E*, S5, *E* and *F*). Notably, while complete knockout of genes in these categories would likely have been lethal (38) (https://depmap.org/portal), inhibition with dCas9-KRAB maintains cell viability and demonstrates the necessity of the targeted genes and protein products for cell surface CD61 expression. Additional gene targets enriched in the CD61^low^ group (genes required for CD61 expression) were chosen for further validation, prioritizing nonessential genes with high expression in Ocy454 cells as determined from mRNA-sequencing of this cell line: *Fhl3*, *Astn1*, *Srf*, *Ndrg4*, and *Clip2* (Fig. 3*C* inset, Data S1 and S2).

### Validation of selected candidates identified in CRISPRi screen *via* shRNA knockdown

**Figure 4:**
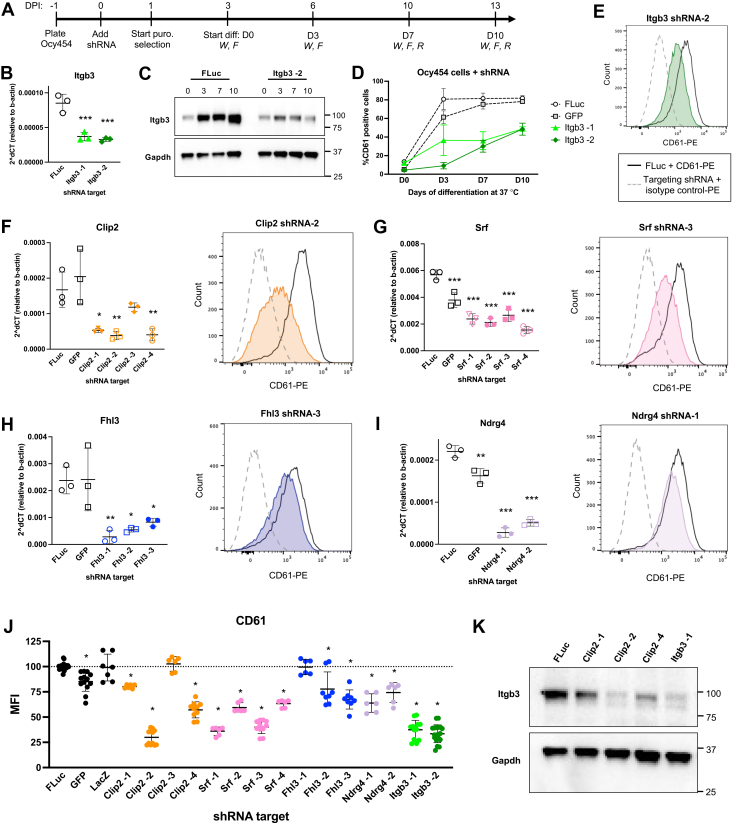
**Confirmation of CRISPRi screening hits using shRNA.***A*, timeline of shRNA infection and analysis. Ocy454 cells were infected with shRNA-containing lentivirus and selected with puromycin (puro). Three days post-infection (DPI), cells were moved to 37 °C to begin differentiation (D0). At differentiation days 0, 3, 7, or 10, cells were collected for analysis by Western blot (W), flow cytometry (F), or qPCR (R). *B*, mRNA expression of *Itgb3* at differentiation day 10 in Ocy454 cells stably expressing the indicated shRNAs. *Lines* show mean ± SD of three biologic replicates. ∗∗∗*p* < 0.001 by one-way ANOVA followed by Dunnett’s multiple comparisons tests *versus* FLuc. *C*, Western blots for Itgb3 and Gapdh at differentiation days 0, 3, 7 and 10 in cells expressing the indicated shRNAs. Molecular weights are indicated in kDa to the *right* of each panel. *D*, flow cytometry results show percent of single, live cells labeled with CD61-PE antibody compared to isotype control at each differentiation timepoint following stable infection with the indicated shRNAs. Error bars show standard deviation for all replicates at each time point. *E*, histograms show intensity of CD61-PE fluorescence compared to isotype control at differentiation day 7 for populations of single, live Ocy454 cells stably expressing FLuc-targeting shRNA or *Itgb3*-targeting shRNA for one representative sample. *F–I*, plots show mRNA expression of targeted genes at differentiation day 7 in Ocy454 cells stably expressing the indicated shRNAs. *Lines* show mean ± SD of three biologic replicates. ∗*p* < 0.05, ∗∗*p* < 0.01, ∗∗∗*p* < 0.001 by one-way ANOVA followed by Dunnett’s multiple comparisons tests *versus* FLuc. Histograms show intensity of CD61-PE fluorescence compared to isotype control at differentiation day 7 for populations of single, live Ocy454 cells stably expressing FLuc-targeting shRNA or the indicated targeting shRNA for one representative sample. *J*, summary of flow cytometry results showing median fluorescence intensity (MFI) of CD61-PE at differentiation day 7 in populations of single, live cells stably expressing the indicated shRNAs. Each point represents one biologic replicate. *Lines* show mean ± SD for all replicates. ∗*p* < 0.001 by one-way ANOVA followed by Dunnett’s multiple comparisons tests *versus* FLuc. *K*, Western blots for Itgb3 and Gapdh at differentiation day 7 in cells expressing the indicated shRNAs. Molecular weights are indicated in kDa to the *right* of each panel.

We initially used sgRNAs from the screen library to validate screen results in a non-pooled format, however, we noted decreasing dCas9-KRAB activity with serial passage of dCas9-Ocy454 cells over time. These findings led us to use lentiviral-mediated shRNA knockdown as an orthogonal approach to confirm CRISPRi screening findings. We designed at least two independent shRNAs for each target and used shRNA sequences targeting firefly luciferase (FLuc), GFP, and LacZ as controls. Using shRNAs targeting *Itgb3*, we observed robust repression of *Itgb3* mRNA, Itgb3 protein, and CD61 surface labeling at 3 to 10 days of differentiation (6–13 days post-shRNA infection) (Fig. 4, *A*–*E*). Likewise, *Eif3b*-targeting shRNAs reduced *Eif3b* mRNA, eIF3B protein, and CD61 surface labeling (Fig. S6, *A*–*C*), together validating the use of shRNAs for knockdown in these multi-week experiments.

With *Eif3b* being a common essential gene, we focused on additional gene targets with promoter-targeted sgRNAs enriched in CD61^low^ cells. Nearly all shRNAs targeting *Fhl3*, *Srf*, *Ndrg4*, and *Clip2* reduced mRNA expression of the targeted transcript (Fig. 4, *F*–*I*). However, no detectable reduction in *Astn1* expression could be achieved with shRNAs, perhaps due to the relatively low baseline expression of this gene (Fig. S6*D*).

Across multiple independent experiments, shRNAs targeting *Srf*, *Clip2*, and *Itgb3* reduced CD61 labeling compared to non-targeting control shRNAs. Both the percentage of cells labeled with CD61 (compared to isotype control) and the median fluorescence intensity (MFI, normalized to non-targeting shRNAs) were reduced (Figs. 4, *E*–*G*, S6, *E*–*K*). The magnitude of CD61 inhibition within each gene target was generally related to the extent of knockdown induced by each shRNA. shRNAs targeting *Fhl3* and *Ndrg4* reduced MFI but not percentage of CD61-positive cells, likely reflecting a more modest overall effect size (Figs. 4, *H* and *I*, S6, *L* and *N*). Overall, the majority of shRNAs targeting each candidate gene significantly reduced CD61 labeling intensity, validating CRISPRi screening results (Fig. 4*J*). Additionally, we confirmed reduction of Itgb3 protein following *Clip2* knockdown by Western blotting with an independent antibody (Fig. 4*K*).

### Genes that control surface CD61 levels also impact mature osteocyte gene expression

**Figure 5:**
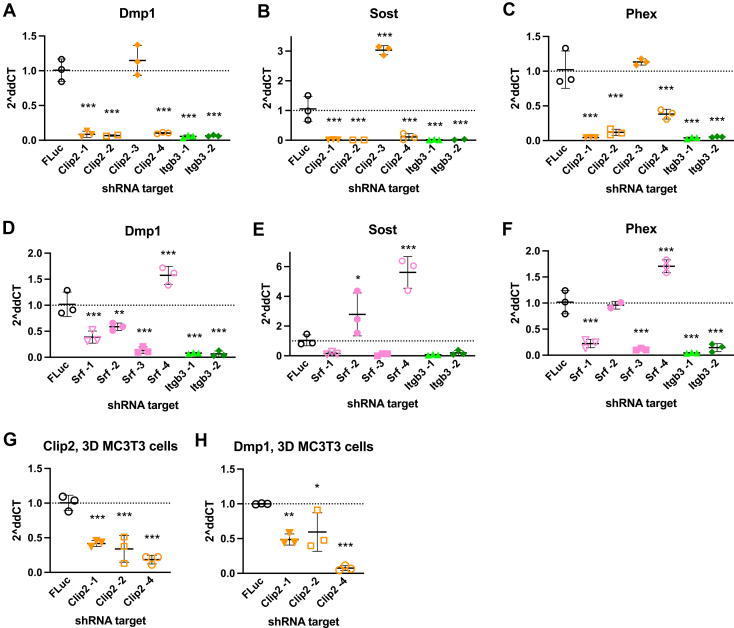
**Genes required for CD61 expression are required for osteocyte maturation.***A–C*, mRNA expression of osteocyte maturity genes at differentiation day 10 in Ocy454 cells stably expressing shRNAs targeting *Clip2* or *Itgb3*. Each point represents one biologic replicate. *Lines* show mean ± SD. ∗∗∗*p* < 0.001 by one-way ANOVA followed by Dunnett’s multiple comparisons tests *versus* FLuc. *D–F*, mRNA expression of osteocyte maturity genes at differentiation day 10 in Ocy454 cells stably expressing shRNAs targeting *Srf* or *Itgb3*. Each point represents one biologic replicate. *Lines* show mean ± SD. ∗*p* < 0.05, ∗∗*p* < 0.01, ∗∗∗*p* < 0.001 by one-way ANOVA followed by Dunnett’s multiple comparisons tests *versus* FLuc. *G* and *H*, mRNA expression of *Clip2* and *Dmp1* in MC3T3-E1 cells stably expressing shRNAs targeting *Clip2* after 6 days of culture in a 3D collagen gel. Each point represents one biologic replicate. *Lines* show mean ± SD. ∗*p* < 0.05, ∗∗*p* < 0.01, ∗∗∗*p* < 0.001 by one-way ANOVA followed by Dunnett’s multiple comparisons tests *versus* FLuc.

Next, we assessed the impact of reducing genes that control CD61 levels on other indices of osteocyte maturation. shRNAs targeting *Srf*, *Clip2*, *and Itgb3* reduced expression of osteocyte differentiation markers *Dmp1, Phex,* and *Sost* in Ocy454 cells (Fig. 5, *A*–*F*). As with CD61 expression, *Clip2* shRNA knockdown efficiency correlated with the measured phenotype; *Clip2* shRNA-3 was less effective at reducing target mRNA levels and did not reduce osteocyte differentiation gene expression. Also consistent with their more modest effect on CD61, *Ndrg4* and *Fhl3* knockdown did not consistently reduce *Sost* or *Dmp1* expression (Fig. S7, *A*–*D*).

Given a previously defined role for *Srf* in bone mineralization *via* osteoblast lineage cells (39), we focused our efforts on *Clip2*. To confirm our findings in a separate system, we studied MC3T3-E1 osteoblastic cells, which take on an osteocyte-like transcriptome and morphology when cultured in 3D collagen gels. MC3T3-E1 cells stably expressing *Clip2*-targeting shRNAs expressed significantly less *Dmp1* after 6 days of culture in 3D collagen gels than FLuc shRNA-expressing cells (Fig. 5, *G* and *H*). Together, these results suggest that *Clip2* expression is required for expression of the mature osteocyte transcriptome.

### Clip2 knockdown affects Ocy454 dendrite formation

**Figure 6:**
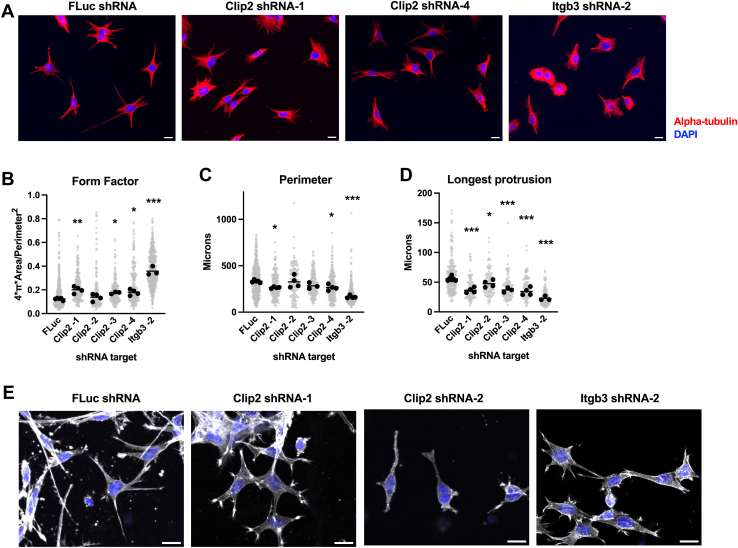
**Clip2 knockdown inhibits Ocy454 dendrite formation.***A*, alpha-tubulin immunofluorescence (*red*) and DAPI (*blue*) in Ocy454 cells stably expressing the indicated shRNAs. Scale bars = 20 μm. *B* and *C*, CellProfiler measurements of form factor and perimeter using alpha tubulin immunofluorescence images. Each *black point* represents the median of all cells in one experimental replicate. *Grey points* show measurements of all individual cells in all replicates. *Lines* designate the mean of n = 3 to 4 experimental replicates. ∗*p* < 0.05, ∗∗*p* < 0.01, ∗∗∗*p* < 0.001 by mixed-effects analysis grouped by experiment, followed by Dunnett’s multiple comparisons tests *versus* FLuc. *D*, quantification of protrusion length for the single longest dendrite of each cell using alpha tubulin immunofluorescence images. Each *black point* represents the median of all measurements in one experimental replicate. *Gray points* show measurements of all individual cells in all replicates. *Lines* designate the mean of n = 3 to 4 independent experimental replicates. ∗*p* < 0.05, ∗∗*p* < 0.01, ∗∗∗*p* < 0.001 by mixed-effects analysis grouped by experiment followed by Dunnett’s multiple comparisons tests *versus* FLuc. *E*, MC3T3 cells labeled with phalloidin (*gray*) and DAPI (*blue*) following stable expression of the indicated shRNAs and 6 days of culture in 3D collagen gels. Images show maximum intensity projections of Z-stacks. Scale bars = 20 μm.

CLIP-115, the protein encoded by *Clip2*, is a microtubule binding protein (40, 41). Therefore, we next visualized the microtubule network in Ocy454 cells using alpha-tubulin immunocytochemistry. Ocy454 cells without shRNA or infected with non-targeting FLuc shRNA displayed extensive tubulin structures in the cell body and dendrites (Figs. 6*A*, S7*E*). While Ocy454 cells infected with FLuc shRNA have numerous long dendrites with asymmetric distribution, cells expressing *Clip2*-targeting shRNAs have shorter projections and more round appearance (Fig. 6*A*). The overall morphology was quantified as increased (more circular) form factor, reduced perimeter, and reduced maximum projection length, consistent with knockdown of *Itgb3* itself (Fig. 6, *B*–*D*). Ocy454 cell morphology visualized with F-actin staining was also affected by *Clip2* and *Itgb3* knockdown, with significantly increased form factor and reduced perimeter (Fig. S7, *F*–*H*) (32). Notably, these data suggest that *Itgb3*/CD61 is both a marker and a regulator of osteocyte differentiation.

Finally, we visualized MC3T3-E1 morphology in 3D collagen gels following *Clip2* knockdown. In this system, MC3T3-E1 cells infected with FLuc shRNA display numerous long dendrite-like cytoskeletal processes. Following *Clip2* or *Itgb3* knockdown, MC3T3 morphology is qualitatively less complex, with fewer and shorter projections (Fig. 6*E*).

In summary, a genome-wide CRISPRi screen focused on surface CD61 expression reveals genes with critical regulatory functions in osteocyte differentiation and morphology and demonstrates a role for the microtubule cytoskeleton to drive osteocyte differentiation.

## Discussion

Here we developed an osteocyte maturation assay that allows high-throughput genetic perturbation analysis of the osteocyte differentiation phenotype. CD61 (integrin β3) is identified as a cell surface marker that tracks with differentiation in Ocy454 cells and serves as the basis of a genome-wide CRISPR interference screen. We identify multiple genes required for full surface expression of CD61, including *Clip2*, which is also required for expression of osteocyte differentiation markers and the dendritic osteocyte phenotype. While typically described as actin-based structures, osteocyte dendrites also contain microtubules, which participate in intracellular trafficking (42), cytoskeletal tension (14), and osteocyte mechanotransduction (13, 15). Proper maintenance of these mechanisms may also impact other aspects of gene expression, differentiation, and morphology. Together, this work suggests that the microtubule cytoskeleton plays an important role in osteocyte differentiation and morphology.

While many other cell surface markers associated with osteocyte maturity were considered as the readout for our screen, the use of CD61 as a readout was supported by scientific and technical factors. Ideal genome-wide screens require thousands of cells containing each sgRNA to be input into the final sorting of high/low populations (43). With time required for sorting as a major bottleneck in the workflow, we required a screening readout that labeled the majority of healthy, mature cells and offered a wide dynamic range for detecting sgRNAs that caused reduced surface marker intensity. In our experiments, antibodies targeting CXCR7, CA-IX, PTGER4, and CD200 labeled less than 10% of differentiated Ocy454 cells, and CD43 expression lacked the appropriate dynamic range over Ocy454 differentiation time. In contrast, CD61 offered practicality as an assay readout with a large proportion of mature cells labeled and a wide dynamic range. Itgb3 is expressed on osteocyte dendrites *in vivo* (29, 30), and Dmp1-mediated deletion of Itgb3 causes osteocytes to form with fewer, shorter dendrites (32), making Itgb3/CD61 a physiologically relevant choice for measuring effects on osteocyte maturity and the dendritic phenotype. Furthermore, osteocyte Itgb3 also supports bone formation and mechanosensation in mice (31, 32), suggesting that CRISPR screening hits affecting CD61 may also have physiological consequences for bone.

CRISPR screening is an established functional genomics approach to identify gene networks that control distinct cellular functions. Here we chose the dCas9 system in a FACS-based cell differentiation screening readout to avoid potential toxicities of Cas9-induced double strand DNA breaks. Therefore, our screen could capture genes that, when inhibited, interfere directly with *Itgb3* transcription, CD61 translation, or CD61 transport to the cell surface. In addition, since surface CD61 expression is associated with the overall degree of osteocyte maturation, genes that reduce cell surface CD61 levels may also generally participate in other aspects of osteocyte differentiation or general mRNA/protein processing. The latter was apparent in the top Gene Ontology terms enriched in the CD61^low^ group: gene targets involved generally in ribosome assembly and translation. Since our screen compared two sorted populations of sgRNA-expressing cells, it should not identify genes whose inhibition leads to lethality, which should drop out of all live populations sorted by flow cytometry. We also anticipated that inhibition of most genes would have minimal effect on CD61 and yet identified 202 gene targets significantly enhanced in CD61^low^ cells.

We selected genes for further study based first on enrichment in CD61^low^ cells, and then on their level of mRNA expression in prior Ocy454 cell RNA-seq data and other bone-relevant datasets. *Astn1*, while one of the most significantly enriched gene targets in the screen, proved challenging to study due to low expression levels and a lack of efficient shRNA sequences. Other putative screening “hits” included *Fhl3* (binds actin and regulates BMP signaling) (44, 45), *Srf* (transcription factor that responds to actin dynamics) (46), *Ndrg4* (expressed in neurons and in cancer, controls β1-integrin clustering) (47, 48), and *Clip2* (microtubule binding protein). Follow-up studies with shRNA knockdown of *Clip2* showed robust effects on CD61 expression, mature osteocyte gene expression, and dendritic morphology.

*Clip2*, also known as *Cyln2* and CLIP-115, is predominantly expressed in the brain, particularly in dendrites within the inferior olive, hippocampus, and piriform cortex (40, 49). It is one of 28 genes affected in the heterozygous chromosomal deletion that causes Williams Syndrome (50), a multisystem disorder with symptoms including vascular stenosis, cognitive impairment, atypical facial features, reduced height and bone mineral density, and hypercalcemia (51, 52, 53). While *Clip2* has primarily been studied for its potential contribution to the neuro-cognitive symptoms of Williams Syndrome, mice lacking *Clip2* have reduced body weight and length (49), suggesting a potential functional role for this gene in bone. At the cellular level, CLIP-115 and closely related protein CLIP-170 (*Clip1*) compete for binding at microtubule plus ends, with CLIP-170 being involved in recruiting the dynein-dynactin complex to microtubule plus ends (49, 54). Fibroblasts lacking *Clip2* showed no measurable differences in microtubule growth rates, however, absence of CLIP-115 resulted in greater localization of CLIP-170 at microtubule plus ends (49), which could contribute to an imbalance in minus-end-directed transport. While *Clip1* inhibition in our screen did not significantly enhance CD61 expression, *Clip2* inhibition is sufficient to disrupt the microtubule network and CD61 surface expression. The role of *Clip2* in osteocyte biology *in vivo* represents an important topic for future study.

One important study limitation is our focus on a single osteocyte-like cell line. Primary osteocytes are heterogeneous and unsuitable for use in a genome-wide screen. Ocy454 cells were selected as a model of osteocyte differentiation since they upregulate marker genes faster than IDG-SW3 cells (55), and MLO-Y4 cells were not selected due to lack of sclerostin expression (56). To validate findings, we used MC3T3-E1 cells grown in 3D culture and found that the *Dmp1* induction and dendrite formation associated with osteoblast-osteocyte differentiation are also reduced by *Clip2* knockdown in this cell line. Further study of the function of *Clip2* in osteocytes *in situ* and of the role of *Clip2* in bone development and maintenance will be important areas of future study.

Early studies of the osteoblast to osteocyte transition visualized microtubules in osteocytes but concluded that they are only localized in the proximal portion of dendrites and that short-term microtubule depolymerization (60–90 min) does not affect osteocyte morphology (11, 12). These observations contrast with those made for actin; actin and actin-binding proteins are found throughout osteocyte dendrites, and short-term actin depolymerization causes dendrite retraction (11, 12, 57), leading many to focus on the role of actin in these structures (58). However, we and others observe microtubules throughout osteocyte dendrites *in vitro* and *in situ* (14, 42), and potential roles for microtubules in initiation of dendrite formation and long-term dendrite stability remain largely unexplored. Independent of dendrite macrostructure, microtubules may be required for proper intracellular trafficking (42, 59, 60) and maintenance of mechanosensitive structures (14, 15, 61) to regulate osteocyte function. Furthermore, microtubule modifications (62, 63) and microtubule-binding proteins (64, 65) can regulate interactions with actin and intermediate filaments, cytoskeletal tension, and cell motility, making additional roles of microtubules and microtubule-binding proteins in osteocytes likely.

In summary, we have used a novel CD61-based Ocy454 cell differentiation assay to identify *Clip2* in a genome-wide CRISPR interference screen as an essential contributor to osteocyte morphology and maturation.

## Experimental procedures

### Cell culture

Ocy454 osteocyte-like cells (28, 66) were cultured in MEM α supplemented with 10% FBS and 1% antibiotic/antimycotic (Gibco) in a 33 °C incubator with 5% CO_2_. At confluence, cells were transferred to a 37 °C incubator to induce differentiation *via* inactivation of the thermosensitive large T antigen. Ocy454 cells were regularly screened for dendritic morphology, marker gene expression, and mycoplasma contamination.

Ocy454 cells were plated at 40,000 cells/ml media the day before lentiviral infection. Fresh media containing 8 μg/ml polybrene and lentiviral particles was added the next day and allowed to incubate overnight. Media was changed the next day to remove the transduction reagents, and then again the next day to begin puromycin selection (4 μg/ml). Cells receiving no lentivirus with or without puromycin were used as infection controls. When puromycin-resistant cells reached confluence, they were transferred to a 37 °C incubator (differentiation day 0).

Sp7 deficient Ocy454 cells stably express a *Sp7*-targeting shRNA (5). HDAC4/HDAC5 double knockout Ocy454 cells stably express a HDAC4-targeting sgRNA and an HDAC5-targeting shRNA (35).

MC3T3-E1 cells were cultured in MEM α supplemented with 10% FBS and 1% antibiotic/antimycotic (Gibco) in a 37 °C incubator with 5% CO_2_. MC3T3-E1 cells were infected with shRNA-containing lentivirus as described and selected with puromycin (4 μg/ml) for at least 5 days. For 3D culture, rat-tail type I collagen (Advanced BioMatrix, 5153) was mixed with neutralization buffer (22 mg NaHCO_3_ per mL of 0.05 N NaOH and 200 mM HEPES) on ice to a pH of 7, then mixed 1:1 with cell suspension. 500 μl cell/collagen mixture was plated into 24-well plates and incubated at 37 °C to allow polymerization. After 10 min, culture media was added to the top of the solidified gel, and cells remained in 3D culture for 5 to 7 days.

### dCas9-KRAB osteocytes

Ocy454 cells were infected with lentivirus containing UCOE-SFFV-dCas9-BFP-KRAB (Addgene #85969) (37) and expanded in culture for 3 days. BFP positive cells were selected by flow cytometry, cultured for another 3 days, and re-sorted for BFP. The resulting polyclonal cell population was used for all sgRNA experiments.

### shRNA and sgRNA preparation

CRISPRi sgRNA sequences and shRNA sequences were designed using Broad Institute tools CRISPick (https://portals.broadinstitute.org/gppx/crispick/public) and TRC shRNA Design (https://portals.broadinstitute.org/gpp/public/seq/search). All sgRNA and shRNA sequences are available in Data S4. sgRNAs were ligated into pLentiGuide (Addgene #117986) and shRNAs were ligated into pLKO.1 (Addgene #10878) using standard protocols, and clones validated by Sanger sequencing.

### Lentivirus preparation

HEK293T cells were cultured in DMEM supplemented with 10% FBS and 0.1% antibiotic/antimycotic in a 37 °C incubator with 5% CO_2_. Cells were plated into 6-well tissue culture plates at 440,000 cells/well. The next day, cells were transfected with viral packaging plasmids psPAX2 and pMD2.G (Addgene #12260, #12259) and insert plasmids containing a single sgRNA or shRNA using PolyJet reagent (SignaGen Laboratories SL100688). Lentivirus was collected for 48 h in media containing 20% FBS, then stored in aliquots at −80 °C.

### mRNA isolation

Ocy454 cells were plated into 24-well plates, individually infected with lentivirus, and differentiated at 37 °C for 7 days. Cells were then washed in PBS and lysed in guanidine-isothiocyanate lysis buffer containing 1% 2-mercaptoethanol. Lysates were passed through QIAshredder columns (Qiagen), then purified with PureLink RNA columns (Invitrogen) according to manufacturer’s instructions. MC3T3-E1 cells cultured in 3D gels were lysed in TRIzol reagent (Invitrogen) and RNA isolated according to manufacturer’s instructions.

### qRT-PCR

Total RNA was reverse transcribed to cDNA with PrimeScript RT Reagent Kit (Takara, including gDNA digestion step). qPCR was performed using SYBR green (Quantabio) with CT values normalized to *b-actin*. All primer sequences are in Data S4.

### Flow cytometry

Ocy454 cells at the specified differentiation timepoint were collected with 0.5% Trypsin-EDTA, washed in FACS buffer (2% FBS in PBS), and resuspended in FACS buffer at approximately 1,000,000 cells/ml. Cells were incubated in FACS buffer at 4 °C for 30 min. Individual reactions were prepared containing approximately 100,000 cells each with 0.5 μl of anti-CD61 (Invitrogen 12-0611-82) or isotype control (Invitrogen 12-4888-83) antibody and incubated at 4 °C for 45 min with gentle rocking. Cells were then washed in FACS buffer, resuspended in FACS buffer, and labeled with SYTOX green nucleic acid stain (Invitrogen #S7020) before flow cytometry. Flow cytometry analysis was primarily performed on an Attune NxT flow cytometer, with stop conditions set to collect 25,000 single, live cells for each replicate.

### Differentiation RNA-seq

Ocy454 cells were differentiated for 1, 7, 10, or 14 days at 37 °C, then collected for RNA in biological duplicates, as described. Total RNA was used for library preparation and sequencing (BGI DNBseq platform). STAR aligner was used to map sequencing reads to transcripts in the mouse reference genome (67). Read counts for individual transcripts were produced with HTSeq-count (68), followed by estimation of expression values using EdgeR. Differential expression analysis was performed using EdgeR after normalizing read counts and including only genes with count per million (CPM) reads >1 for at least one sample. RNA sequencing data have been deposited at NCBI’s Gene Expression Omnibus under accession number GSE311295.

### CD61 RNA-seq

Ocy454 cells were differentiated for 17 days at 37 °C. Three independent groups of cells were then collected and prepared for CD61 flow cytometry as described. The top 10% and bottom 10% of live CD61-PE-labeled cells were collected into FACS buffer containing RNase inhibitor. Cells were then pelleted and lysed for RNA collection as described. Lysates were passed through QIAshredder columns, then total RNA was isolated using the Arcturus PicoPure RNA isolation kit (Applied Biosystems) with on-column DNase (QIAgen). Total RNA was used for library preparation and sequencing (BGI DNBseq platform).

At least 18 million paired-end reads per sample were obtained from PolyA-selected libraries, with >89% of reads mapping uniquely to the mouse transcriptome (GRCm39 Release 104; Salmon) (69). Transcript counts were collapsed to gene counts, and differential expression analysis was performed with DESeq2 (version 1.42.1; R version 4.3.1) (70). Genes with at least 5 normalized counts in all samples were considered for analysis. RNA sequencing data have been deposited at NCBI’s Gene Expression Omnibus under accession number GSE311295.

### CRISPRi screen

The Dolomiti A lentivirus library (71) containing 67,366 sgRNAs targeting promoters of mouse genes and control sequences (approximately 3 sgRNAs/gene target) was purchased from the Broad Institute. Viral titer was initially determined by measuring puromycin resistance following serial dilutions of virus in dCas9-Ocy454 cells. Two independent replicates of the screen were performed. 24 million cells in 15 15-cm tissue culture dishes were infected at target MOI 0.3 to 0.5 in MEM-alpha media containing 8 μg/ml polybrene (Sigma H9268). On day 2 post-infection, sgRNA-containing cells were selected with puromycin (4 μg/ml). At confluence, puromycin-resistant cells were transferred to 37 °C to induce differentiation. On differentiation day 10, cells were collected in three batches and labeled with CD61-PE and SYTOX-Green as described above. Sorting was performed on a FACSAria (Becton Dickinson) to collect the top 10% and bottom 10% of the live CD61-PE-labeled population. Sorted cells were collected into 100% FBS prior to genomic DNA isolation.

### CRISPRi screen analysis

Cell pellets from each group were stored at −80 °C, then genomic DNA (gDNA) was extracted with the NucleoSpin Blood Kit (Machery Nagel) with the following modifications from the manufacturer’s instructions: lyse cell pellets overnight, then treat with RNase A for 5 min. After DNA precipitation and washing, further purify the gDNA with PCR inhibitor removal kit (Zymo #D6030). sgRNA sequences were PCR amplified from gDNA using Titanium *Taq* DNA Polymerase. Four replicate reactions per sample, each containing 10 μg gDNA, were amplified with 32 PCR cycles. Primers added barcodes for each reaction and Illumina sequencing adaptors. PCR products were purified, pooled, and sequenced in one lane of a HiSeq-2500 (Illumina).

Reaction barcodes and sgRNAs were parsed from each sequencing read using PoolQ (version 2.2), then counts were assigned to known library sgRNAs without allowing mismatches. Total sgRNA counts for all reactions for each sample were then aggregated. Target-level aggregation, fold change calculations, and statistical analysis for both replicates were performed with Apron (version 1.0) for all gene targets with at least three sgRNAs. Non-targeting and intergenic site-targeting sgRNAs were grouped into 3-guide pseudogenes for points plotted as controls.

### Western blots

Cells expressing shRNAs were washed in cold PBS, pelleted, and lysed using TNT protein lysis buffer (20 mM Tris pH 8, 200 mM NaCl, 0.5% Triton X-100) supplemented with NaF, vanadate, DTT, and protease inhibitor cocktail (Selleck USA). Clarified proteins were separated on 8% SDS-PAGE gels, blocked, and labeled overnight with antibodies against eIF3B (Bethyl A301–760A), Itgb3 (Cell Signaling #13166), or Gapdh (Cell Signaling #2118), with antibody specificity verified by shRNA knockdown and molecular weight. Blots were then incubated with HRP-linked anti-rabbit secondary, developed with ECL Plus (Thermo Scientific Pierce), and imaged on an iBright Imaging System (Invitrogen).

### Immunofluorescence

Ocy454 cells were plated into 12-well plates, infected with shRNA lentivirus, and selected with puromycin (4 μg/ml) for at least 5 days. Ocy454 cells stably expressing shRNAs were then plated into chamber slides at uniform density and allowed to attach for at least 2 days. Cells were fixed in pre-warmed 4% PFA for 15 min, washed in PBS, and permeabilized in 0.5% Triton X-100 in HBSS. F-actin staining (Abcam Cytopainter Green) was performed at room temperature for 1 to 2 h, followed by DAPI labeling.

For alpha tubulin immunofluorescence, permeabilized cells were blocked in 5% BSA in HBSS with 0.1% Triton X-100, then incubated with anti-alpha tubulin (Sigma T9026) or mouse IgG (sc-2025, 2.6 μg/ml final concentration) overnight at 4 °C (14, 72). Secondary antibody (Invitrogen A31570) was applied in 1% BSA in HBSS with 0.1% Triton X-100 at room temperature for 2 h, followed by DAPI labeling. Imaging was performed on a Nikon Eclipse microscope.

### Image analysis

Single-channel images were analyzed with CellProfiler (version 4.2.6) to identify single cells and measure quantitative morphology features (73). Our pipeline segmented DAPI-stained nuclei as primary objects, then used the phalloidin-stained actin cytoskeleton or the alpha tubulin-stained microtubule cytoskeleton to segment individual cells. Form factor and perimeter were automatically measured for each identified cell. Protrusion length from the edge of the nucleus to the end of the alpha tubulin-stained protrusion was measured manually for the longest dendrite of each cell in a blinded manner using ImageJ (74).

### Statistical analysis

With the exception of sequencing-based outcomes, statistical analyses were performed in GraphPad Prism (version 10.0.2). Intensity and proportion of CD61-labeling were compared by one-way ANOVA with Dunnett’s multiple comparisons tests against no sgRNA control or against FLuc-targeting shRNA. qPCR expression of target genes was compared by one-way ANOVA with Dunnett’s multiple comparisons tests against cells infected with FLuc-targeting shRNA. For image analysis, medians were calculated for all measurements in each experimental replicate. Median values within one replicate were treated as paired and compared using a mixed-effects analysis followed by Dunnett’s multiple comparisons tests.

## Data availability

Raw RNA sequencing data have been deposited at NCBI’s Gene Expression Omnibus under accession number GSE311295. RNA sequencing results are contained with the supporting information. CellProfiler pipeline has been archived at cellprofiler.org.

## Supporting information

This article contains supporting information.

## Conflict of interest

The authors declare that they have no conflicts of interest with the contents of this article.
