# Supplementary material for: Genome-wide CRISPR interference screen identifies Clip2 as a novel regulator of osteocyte maturation and morphology
Source: J Biol Chem. 2026 Apr 27;302(6):113075. doi: 10.1016/j.jbc.2026.113075 (PMC13254583; doi:10.1016/j.jbc.2026.113075)
Supplement: Data S1 [file mmc1.pdf]

Genome-wide CRISPR interference screen identifies Clip2 as a novel regulator of osteocyte maturation and morphology

Courtney M. Mazur, Parthena E. Kotsalidis, Majd George, Tom Whalley, Tadatoshi Sato, John G. Doench, Lauren E. Surface, and Marc N. Wein

Supporting Information

Document S1: Supplemental Figures 1-7.

Supplemental Data 1: Table of normalized gene counts and differential expression from Ocy454 differentiation RNA sequencing.

Supplemental Data 2: Table of normalized gene counts and differential expression from CD61 FACS RNA sequencing.

Supplemental Data 3: List of gene targets significantly enriched ($Log_2FC<-1$, $p<0.05$) in the $CD61^{low}$ group of CRISPRi screen.

Supplemental Data 4: Primers and DNA oligos.

Supplemental Figure 1

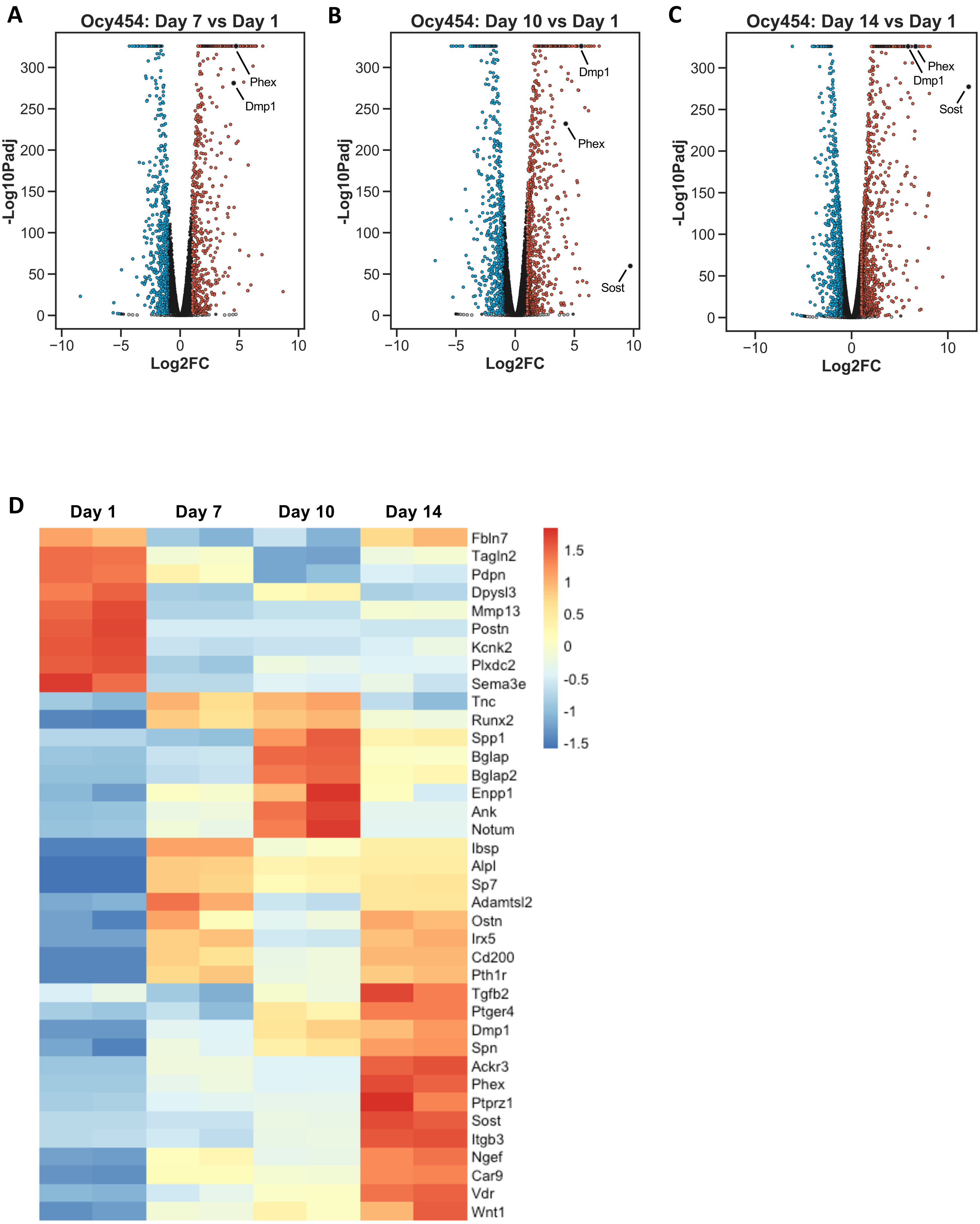

Supplemental Figure 2

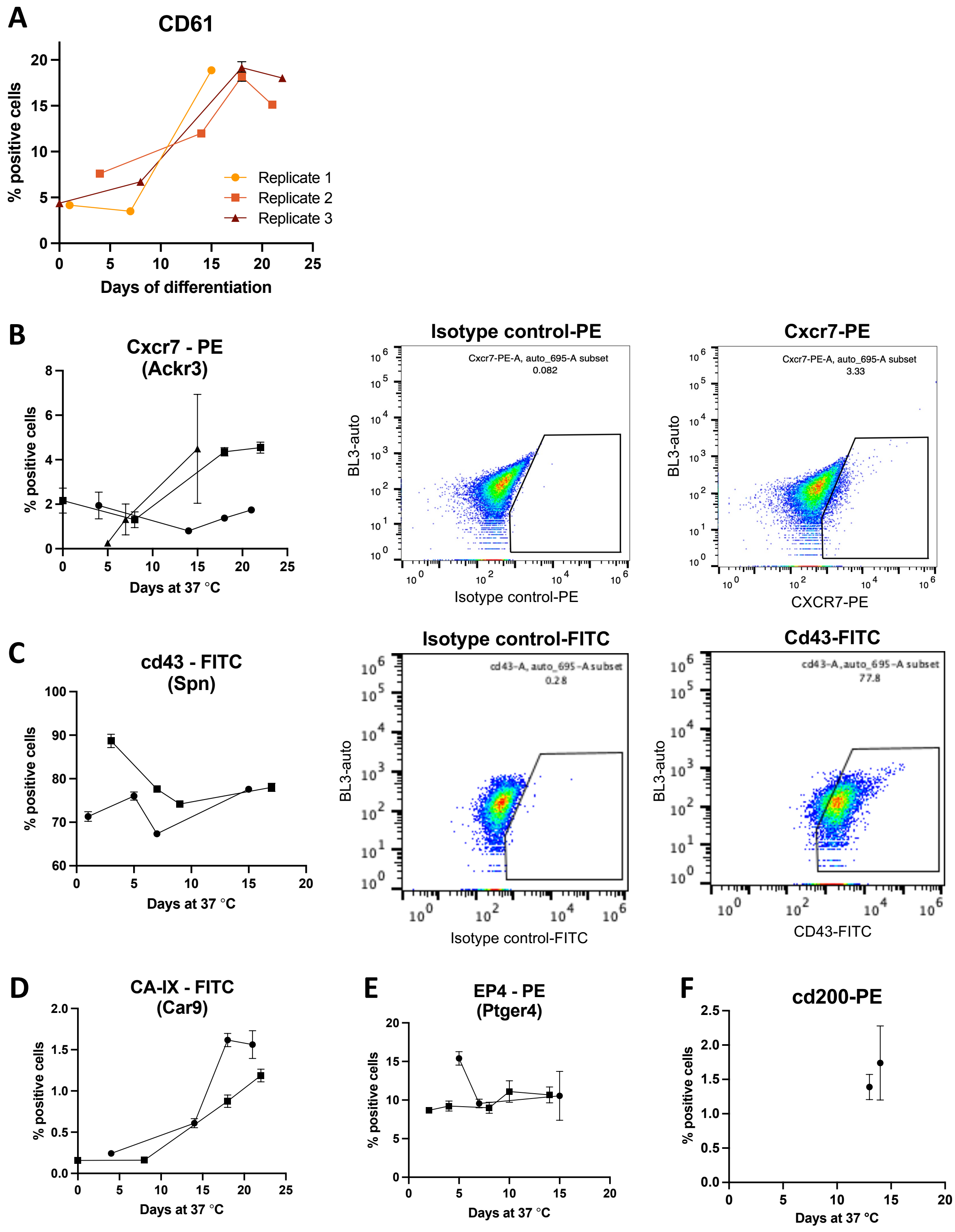

Supplemental Figure 3

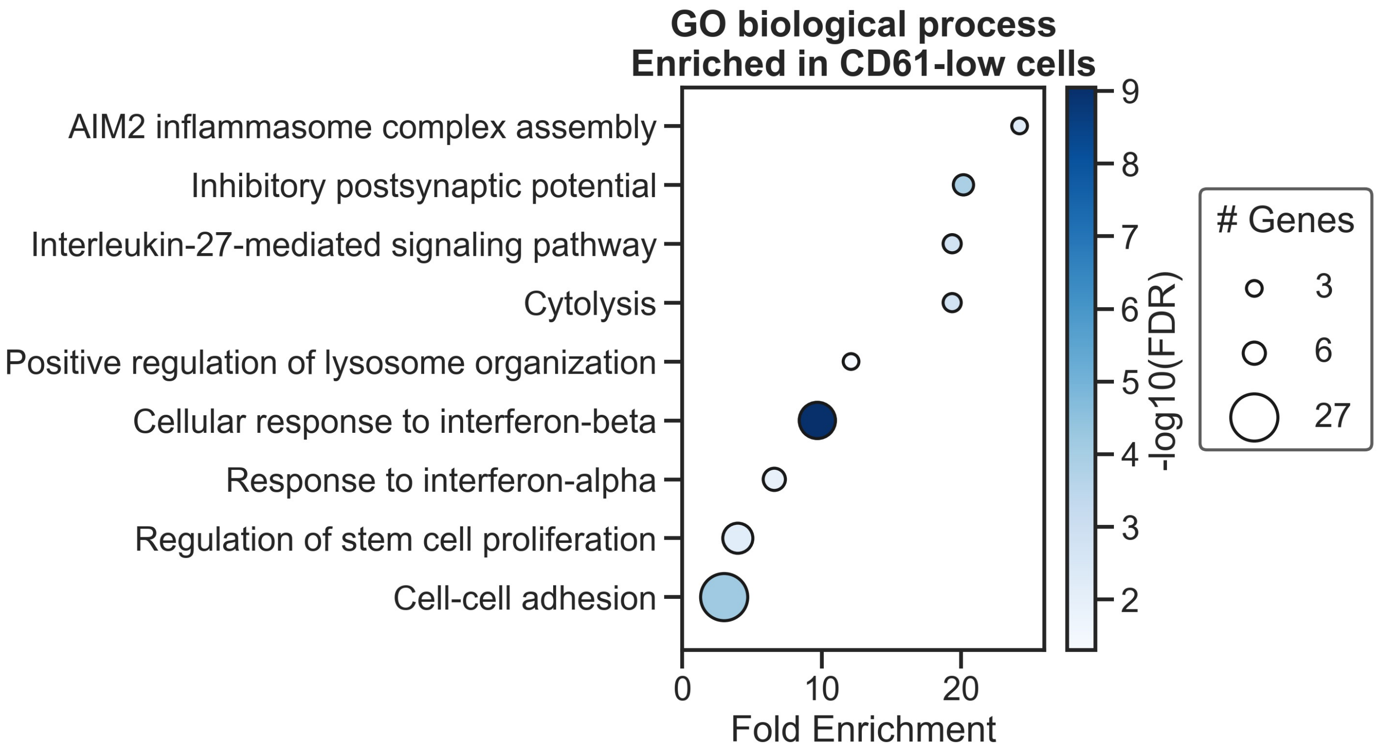

Supplemental Figure 4

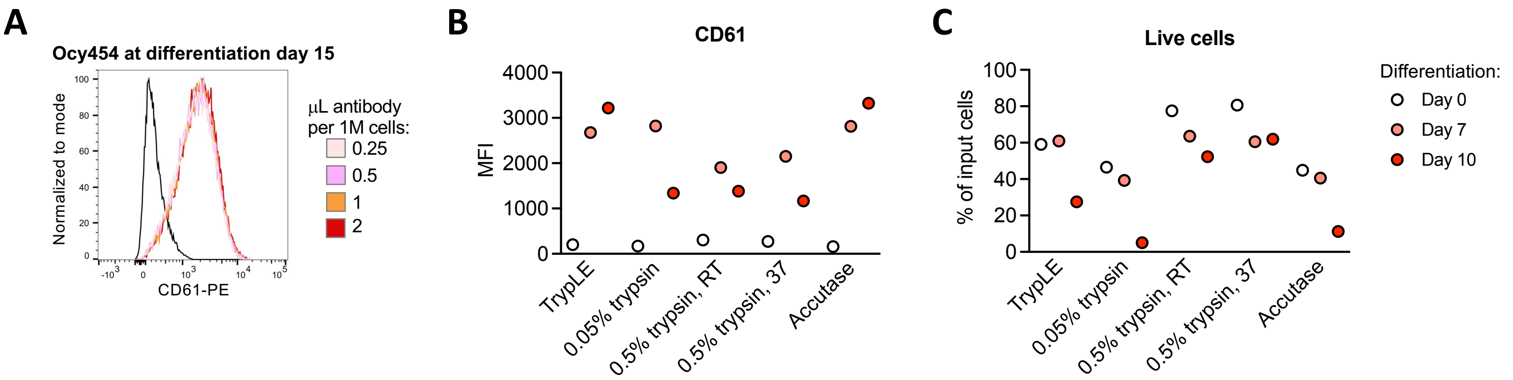

Supplemental Figure 5

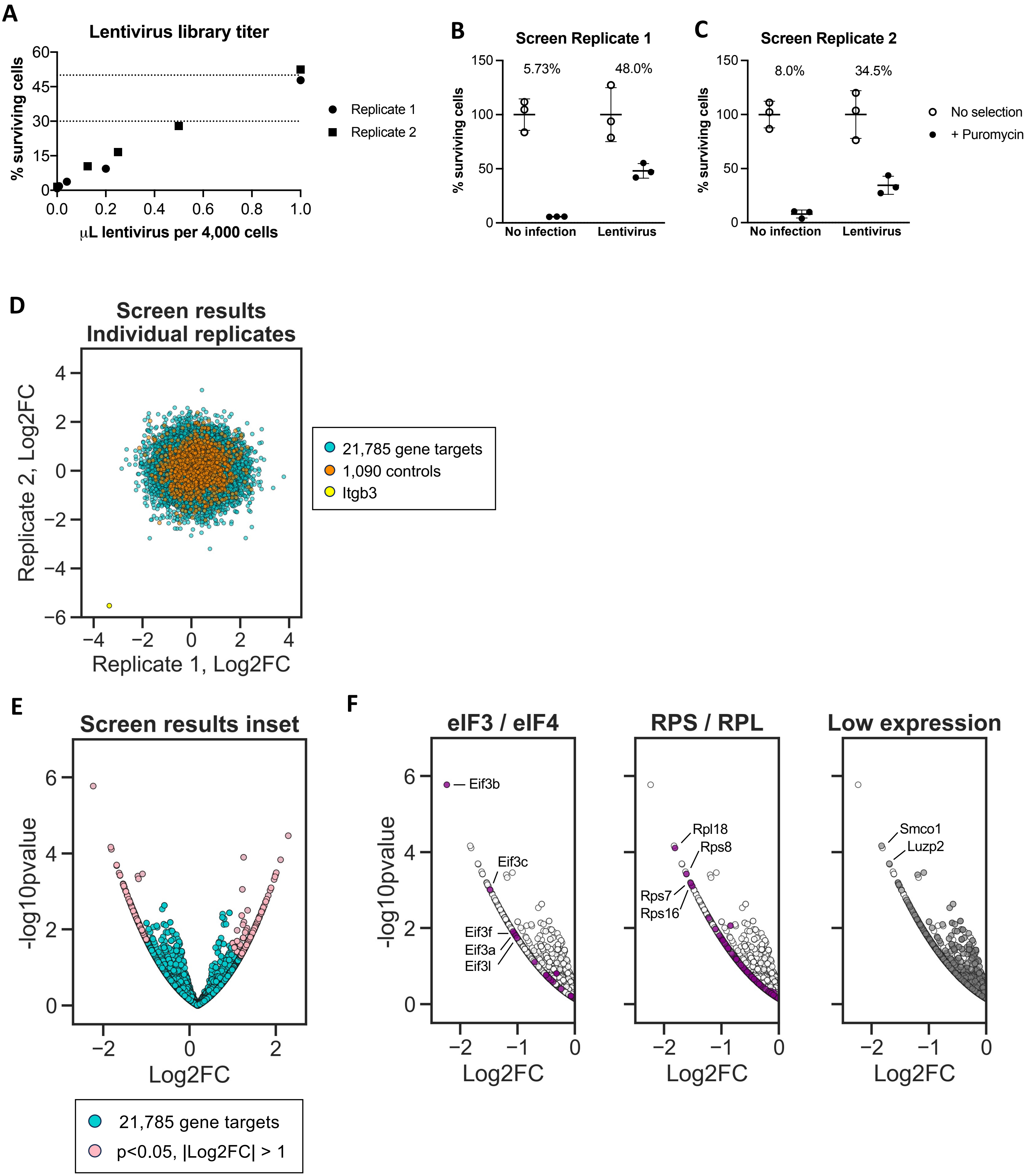

Supplemental Figure 6

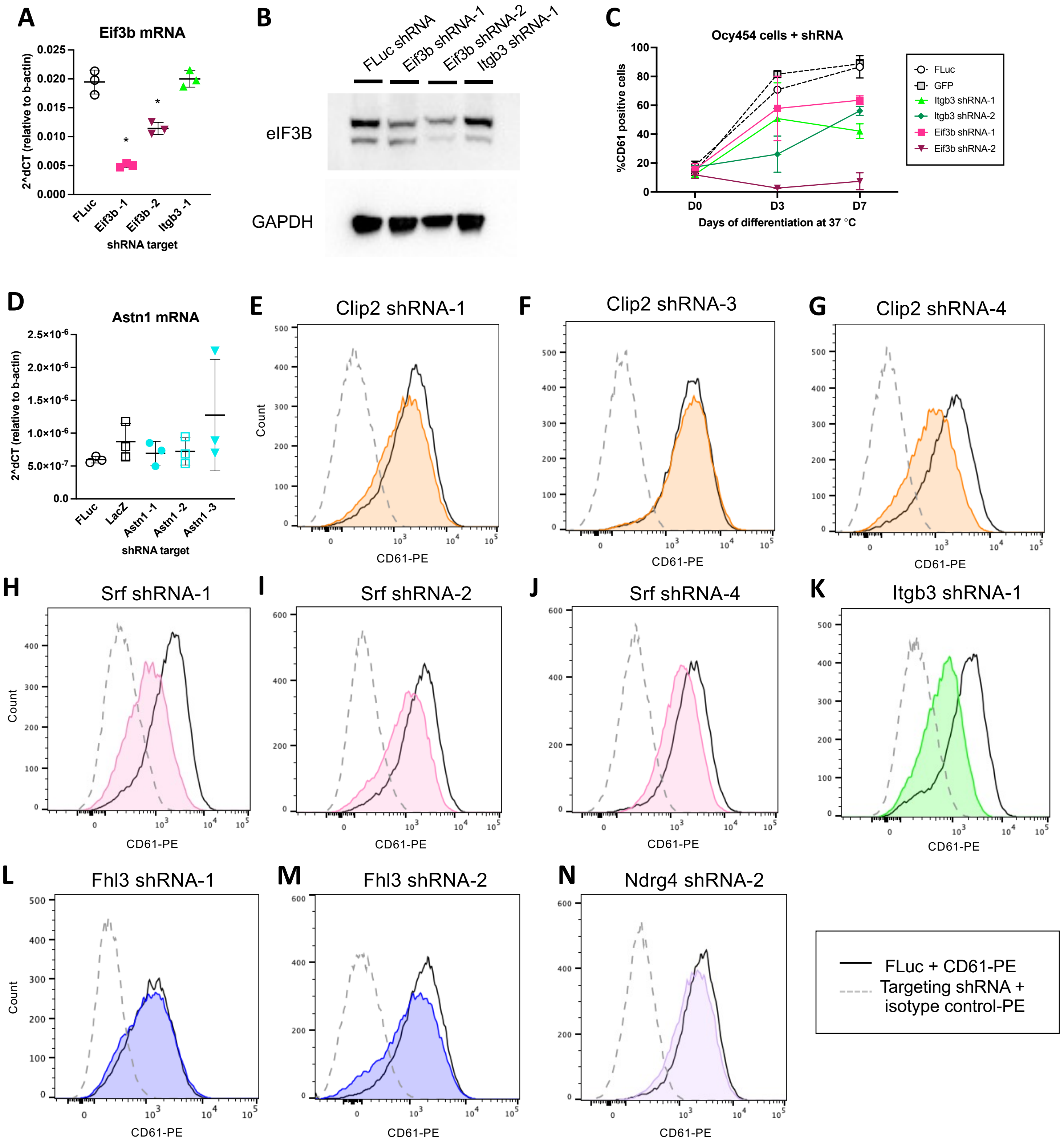

Supplemental Figure 7

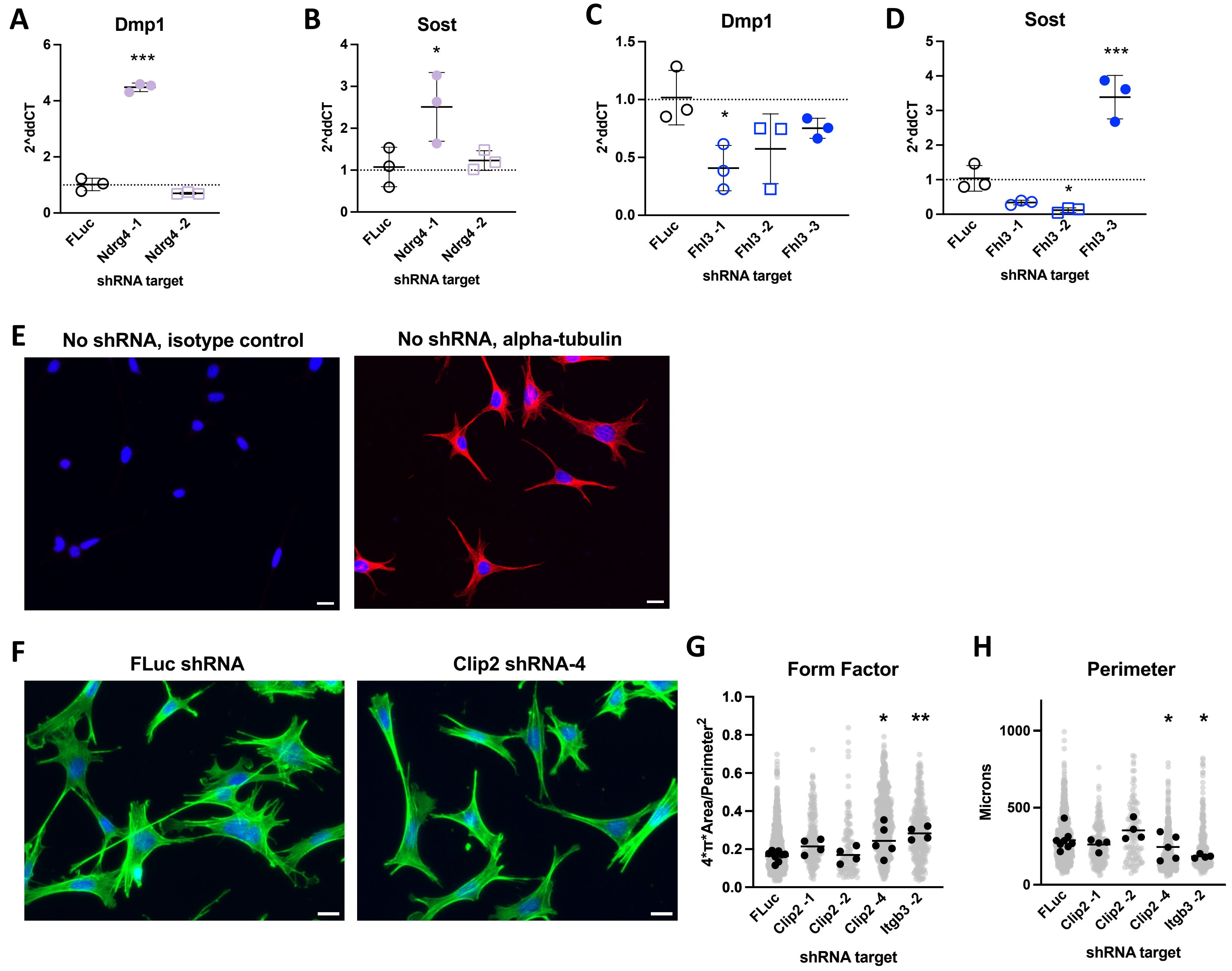

Supplemental Figure Legends

Supplemental Figure 1:
A) Volcano plot of Ocy454 differentiation at day 7 compared to day 1.
B) Volcano plot of Ocy454 differentiation at day 10 compared to day 1.
C) Volcano plot of Ocy454 differentiation at day 14 compared to day 1.
D) Heat map shows z-scored counts per million for osteoblast to osteocyte transition genes, osteocyte signature genes, and genes encoding prospective surface markers for flow cytometry. All genes included in heat map are significantly differentially expressed (FDR<0.01) in at least one comparison to Day 1. Scaling is calculated individually for each row; therefore, no inferences should be made about relative abundances of genes within each timepoint.

Supplemental Figure 2:
A) Flow cytometry results show percent of single, live Ocy454 cells labeled with CD61-FITC antibody compared to isotype control at a range of differentiation timepoints. Lines connect matched subcultures of cells followed over time. Error bars show standard deviation for technical replicates in each test.
B) Flow cytometry results show percent of single, live Ocy454 cells labeled with CXCR7-PE antibody compared to matched isotype controls at a range of differentiation timepoints. Lines connect matched subcultures of cells followed over time. Error bars show standard deviation for technical replicates in each assay. Scatter plots show representative tests of single, live, CXCR7 and isotype control-labeled cells at differentiation day 15.
C) Flow cytometry results show percent of single, live Ocy454 cells labeled CD43-FITC antibody compared to matched isotype control at a range of differentiation timepoints. Lines connect matched subcultures of cells followed over time. Error bars show standard deviation for technical replicates in each assay. Scatter plots show representative tests of single, live, CD41 and isotype control-labeled cells at differentiation day 15.
D-F) Flow cytometry results show percent of single, live Ocy454 cells labeled with indicated antibodies compared to matched isotype controls at a range of differentiation timepoints. Lines connect matched subcultures of cells followed over time. Error bars show standard deviation for technical replicates in each assay.

Supplemental Figure 3: Top gene ontology terms of 501 genes enriched in CD61-low cells ($Log_2FC>1$ and $p_{adj}<0.01$).

Supplemental Figure 4:
A) Histograms show intensity of CD61-PE fluorescence compared to isotype control (black line) in populations of single, live Ocy454 cells when equal numbers of cells were incubated with the indicated amount of total antibody.
B) Median fluorescence intensity (MFI) of CD61-PE following cell dissociation with each reagent at a range of differentiation timepoints. Each point represents one test of >25,000 live single cells.
C) Percent of prepared cells passing FACS gates based on size, singlets, and viability following cell dissociation with each reagent at a range of differentiation timepoints.

Supplemental Figure 5:
A) Scatter plot showing cell viability after infection with Dolomiti A lentivirus library and puromycin selection, indicating multiplicity of infection (MOI) achieved with each volume of lentivirus. The targeted MOI of 30-50% is indicated with horizontal dashed lines.
B) Cell viability after infection with Dolomiti A lentivirus library or no lentivirus and puromycin selection or no puromycin selection in CRISPRi screen replicate 1. Cells were treated in parallel

to the screen and prepared in biologic triplicates. Counts were performed after 5 days of puromycin selection.
C) Cell viability after infection with Dolomiti A lentivirus library or no lentivirus and puromycin selection or no puromycin selection in CRISPRi screen replicate 2. Cells were treated in parallel to the screen and prepared in biologic triplicates. Counts were performed after 4 days of puromycin selection.
D) Scatter plot shows Log2FC results for CD61^{high} vs CD61^{low} in two individual replicates of the CRISPRi screen. Each point represents one gene targeted by at least 3 sgRNAs in the pooled screening library. Control points represent groups of three non-targeting or intergenic site-targeting sgRNAs.
E) Inset of volcano plot showing CRISPRi screen results for CD61^{high} vs CD61^{low} groups for both replicates. Control sgRNAs and Itgb3-targeting sgRNAs are omitted. Each point represents one gene targeted by at least 3 sgRNAs in the pooled screening library.
F) Volcano plot insets showing all gene targets enriched in CD61^{low} cells ($Log_2FC<0$). Labeled genes encode components of the eukaryotic translation initiation factor complex (eIF3/eIF4), encode small or large ribosomal protein subunits (RPS/RPL), or are not detected in RNA-sequencing datasets of Ocy454 cells (Supplemental Figure 1, Figure 1G).

Supplemental Figure 6:
A) mRNA expression of *Eif3b* in Ocy454 cells at differentiation day 7 following stable infection with the indicated shRNAs. Lines show mean +/- SD of three biologic replicates. *p<0.05.
B) Western blot shows expression of eIF3B protein in Ocy454 cells at differentiation day 7 following stable infection with the indicated shRNAs.
C) Flow cytometry results show percent of cells labeled with CD61-PE antibody compared to isotype control at each differentiation timepoint following stable infection with the indicated shRNAs. Error bars show standard deviation for all replicates at each time point.
D) mRNA expression of *Astn1* in Ocy454 cells at differentiation day 7 following stable infection with the indicated shRNAs. Lines show mean +/- SD of three biologic replicates.
E-N) Histograms show intensity of CD61-PE fluorescence compared to isotype control at differentiation day 7 for cells stably expressing the indicated shRNA and cells expressing FLuc shRNA.

Supplemental Figure 7:
A-D) mRNA expression of osteocyte maturity genes at differentiation day 10 in Ocy454 cells stably expressing the indicated shRNAs. Lines show mean +/- SD. *p<0.05, ***p<0.001 by one-way ANOVA with Dunnett's multiple comparisons tests compared to FLuc.
E) Alpha-tubulin immunofluorescence or isotype control immunofluorescence (red) and DAPI (blue) in Ocy454 cells without shRNA infection. Scale bars = 20 μm.
F) Ocy454 cells labeled with phalloidin (green) and DAPI (blue) following stable expression of the indicated shRNAs. Scale bars = 20 μm.
G-H) Cell Profiler measurements of form factor and perimeter using phalloidin staining images. Each plotted point represents metrics of one cell. Each black point represents the median of all measurements in one experimental replicate. Grey points show measurements of all individual cells in all replicates. Lines designate the mean of n=4-8 experimental replicates. *p<0.05, **p<0.01, ***p<0.001 by mixed-effects analysis grouped by experiment followed by Dunnett's multiple comparisons tests versus FLuc.
